# Ocular syphilis masquerading as refractory retinal diseases

**DOI:** 10.1186/s12879-023-08739-2

**Published:** 2024-02-07

**Authors:** Sung Who Park, Kye-Hyung Kim, Han Jo Kwon, Ik Soo Byon, Youan Hasan Khan, Quan Dong Nguyen

**Affiliations:** 1 Department of Ophthalmology, School of Medicine, Pusan National University, Busan, South Korea; 2 Medical Research Institute, School of Medicine, Pusan National University Hospital, Busan, South Korea; 3 Spencer Center for Vision Research, Byers Eye Institute, Stanford University, 2370 Watson Court, Suite 200, Palo Alto, CA 94303 USA; 4 Department of Infection, School of Medicine, Pusan National University, Busan, South Korea

**Keywords:** Age related macular degeneration, Choroidal neovascularization, Ocular syphilis, Syphilis, Diabetic macular edema, Latent syphilis

## Abstract

**Purpose:**

To report two cases of syphilis masquerading as chronic refractory macular diseases.

**Case descriptions:**

Two patients had been diagnosed with neovascular age-related macular degeneration (neovascular AMD) and diabetic macular edema (DME), respectively. The disease worsened despite repeated intravitreal injections of anti-vascular endothelial growth factor (VEGF) and also surgical treatment (in suspected case of DME). Systemic evaluations were positive for syphilis. Intravenous penicillin was started, and the macular diseases improved. The lesions were well controlled afterward.

**Conclusions:**

The current two cases demonstrated that ocular syphilis can masquerade as refractory chronic retinal diseases such as DME and neovascular AMD. Laboratory evaluations for syphilis may be needed, not only for uveitis but also for refractory retinal diseases. Indocyanine green angiography may be helpful to reveal occult syphilis.

## Introduction

The spirochete bacterium *Treponema pallidum* causes infection known as syphilis [1]. Given that it is known as ‘the great imitator’ for its ability to mimic many diseases [2] and its diagnosis is dependent on laboratory findings, screening for syphilis is usually considered a routine test for patients with uveitis and ocular inflammatory diseases [3]. The two cases of ocular syphilis masquerade as refractory chronic macular disease. As they did not show evidence of ocular inflammation on slit lamp examination, fundus examination, fundus photo optical coherent tomography (OCT), and fluorescein angiography (FA), it implies that the screening test for syphilis may be needed not only for cases of uveitis but also for the refractory retinal vascular diseases.

## Case I

**Fig. 1:**
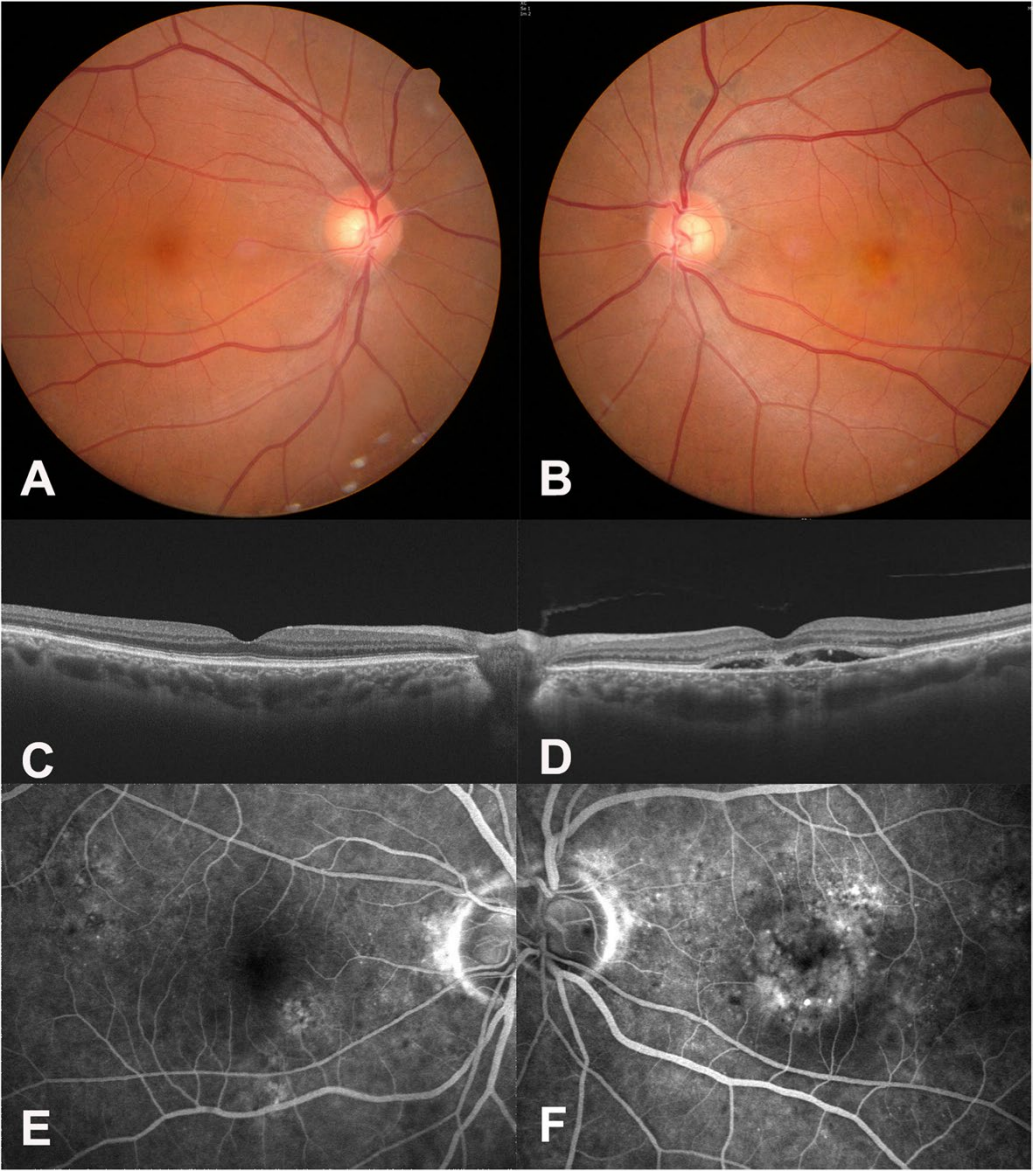
Ocular images at the initial visit of the patient in Case I. **A** No abnormal finding was noted on fundus photograph of the right eye. **B** Small subfoveal hemorrhage was seen on fundus photograph of the left eye. **C** No abnormal finding was noted on optical coherent tomography (OCT) of the right eye. **D** Subretinal fluid was seen on OCT of the left eye. **E** No definite leakage was noted on fluorescent angiography (FA) of the right eye. **F** Macular leakage was noticed on FA of the left eye

A 72-year-old male presented with decreased visual acuity in his left eye (OS) for three months. Best corrected visual acuity was 20/25 in OS. A diagnosis of neovascular age-related macular degeneration (neovascular AMD) was made in OS through fundus photos (Fig. 1B), optical coherence tomography (OCT, Fig. 1D), and fluorescein angiography (FA, Fig. 1F). No drusen was found in either of his eyes (Fig. 1) and no cellular reactions were noticed in both eyes.

**Fig. 2:**
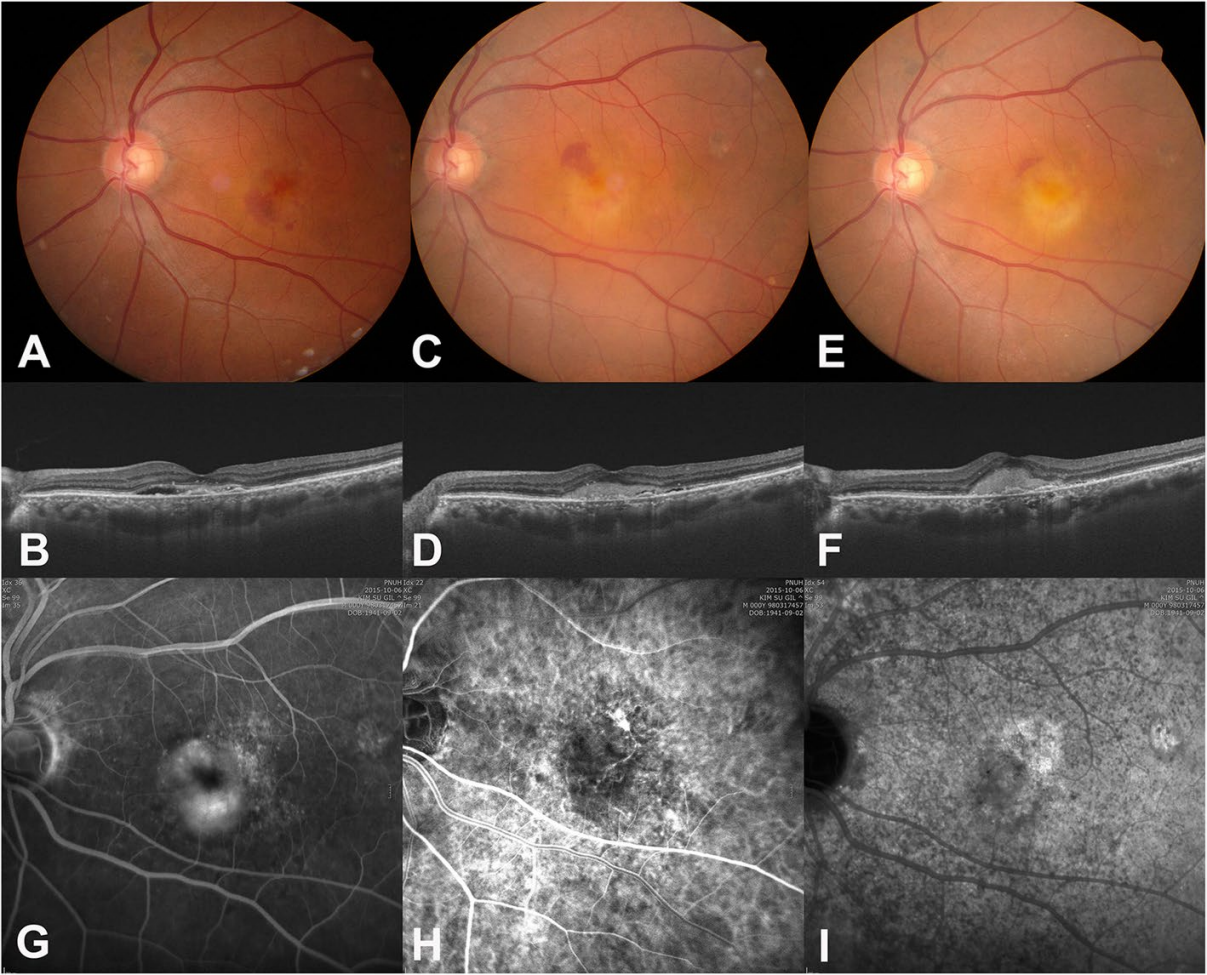
Ocular images after intraocular injections in Case I. **A** Subfoveal hemorrhage increased after two injections of aflibercept on fundus photograph. **B** Subretinal fluid hemorrhage decreased slightly on optical coherent tomography (OCT) after two injections of aflibercept. **C** The fovea turned yellowish in fundus photo after two subsequent injections of ranibizumab and two injections of bevacizumab. **D** Subretinal exudation was noticed on OCT after two injections of ranibizumab and two injections of bevacizumab. **E** The yellowish macular lesion worsened after an intravitreal injection of triamcinolone. **F** Subretinal exudation increased after the triamcinolone injection **G** Fluorescein angiography (five minutes) showed no inflammatory leakage other than choroidal neovascularization (CNV). **H** A CNV was noted on an early phase (one minute) of indocyanine green angiography (ICGA). **I** A late phase (15 min) of ICGA showed multiple hypo-fluorescent dots

Intravitreal injections of aflibercept were administered twice at monthly intervals. Subretinal fluid (SRF) showed mild improvement, while subretinal hemorrhage (SRH) worsened (Fig. 2A and B). Two intravitreal injections of bevacizumab and two of ranibizumab were also administered but were not effective at controlling the maculopathy (Fig. 2C and D). The lesion worsened further despite subsequent treatment with intravitreal triamcinolone (4 mg/0.1 ml) (Fig. 2E and F). Visual acuity of OS fell to 20/200. Additional FA and Indocyanine green angiography (ICGA) were performed. FA showed no specific lesion other than choroidal neovascularization (CNV) (Fig. 2G). CNV was noticed in an early phase of ICGA (Fig. 2H). A late phase ICGA showed multiple hypo-fluorescent dots (Fig. 2I) which were suggestive of inflammation of choriocapillaris. Again, there were no cellular reactions in anterior chamber as well as vitreous.

**Fig. 3:**
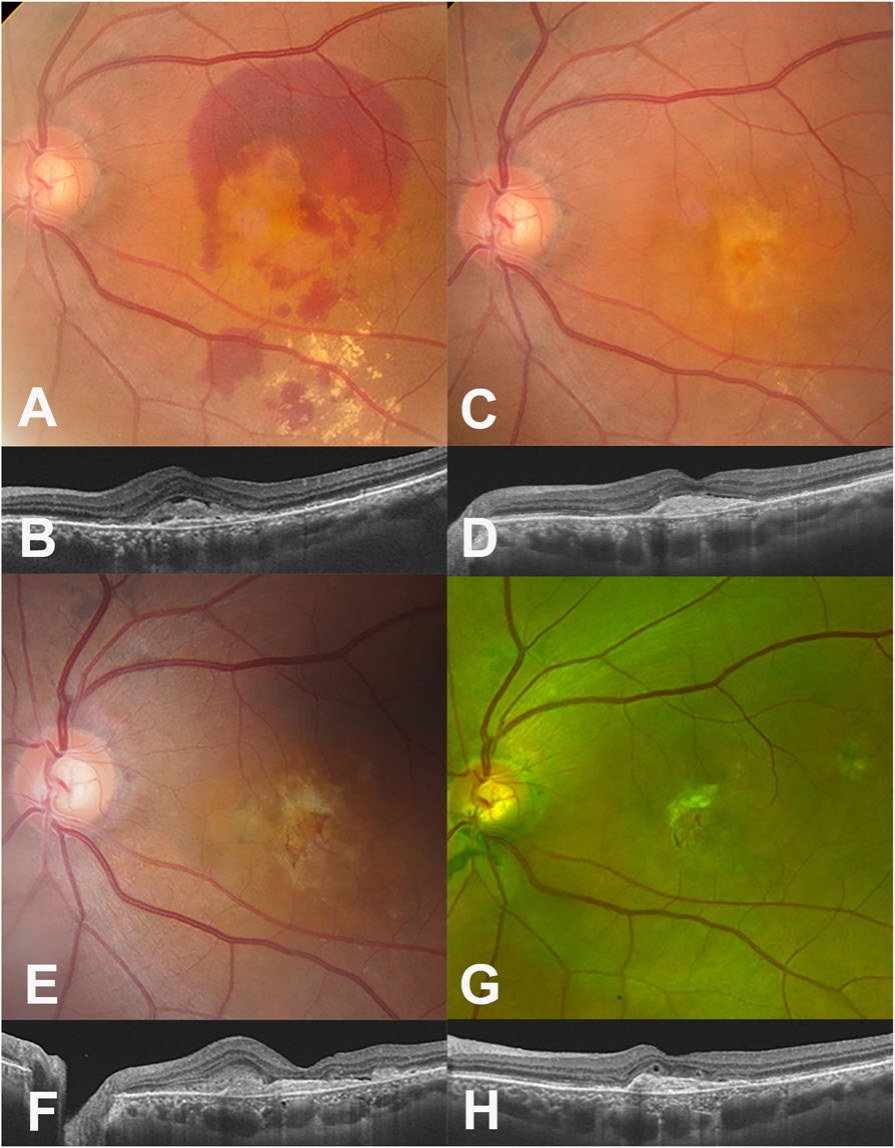
Ocular images after penicillin therapy in Case I. **A** and **B** Macular hemorrhage increased but subretinal infiltration decreased seven days after starting penicillin therapy on optical coherent tomography. **C** and **D** After two ranibizumab injections, macular hemorrhage disappeared, and fibrosis was noted. **E** and **F** Four years later, the CNV was reactivated in the nasal foveal region **G** and **H** The reactivated CNV was well controlled with bevacizumab injections based on a treat-and-extend regimen

Laboratory evaluation was performed to search for factors related to choroidal inflammation (uveitis) which resulted in a positive venereal disease research laboratory (VDRL) test and a positive fluorescent treponemal antibody (FTA -ABS) Immunoglobulin (Ig) G; other evaluations were within normal limits. The patient was referred to an infectious disease specialist, and intravenous penicillin was infused for two weeks. Although the macular hemorrhage increased, the yellowish color on macular nearly disappeared 7 days after starting penicillin infusion. (Fig. 3A and B). One month after starting penicillin infusion, the macular exudation further improved, but some fluid still remained. The titer of VDRL decreased from 1:4 to 1:2. The lesion improved with two additional ranibizumab injections at 6-week intervals (Fig. 3C and D). It had been stable for another four years without any additional treatment. Four years later, the CNV was reactivated in the nasal foveal region (Fig. 3E and F. Given that VDRL titers had remained stable, the patient did not get additional antibiotic therapy. The reactivated CNV was well controlled with bevacizumab injections based on a treat-and-extend regimen (Fig. 3G and H). At the most recent visit, the best corrected visual acuity of the left eye was 20/200.

## Case II

**Fig. 4:**
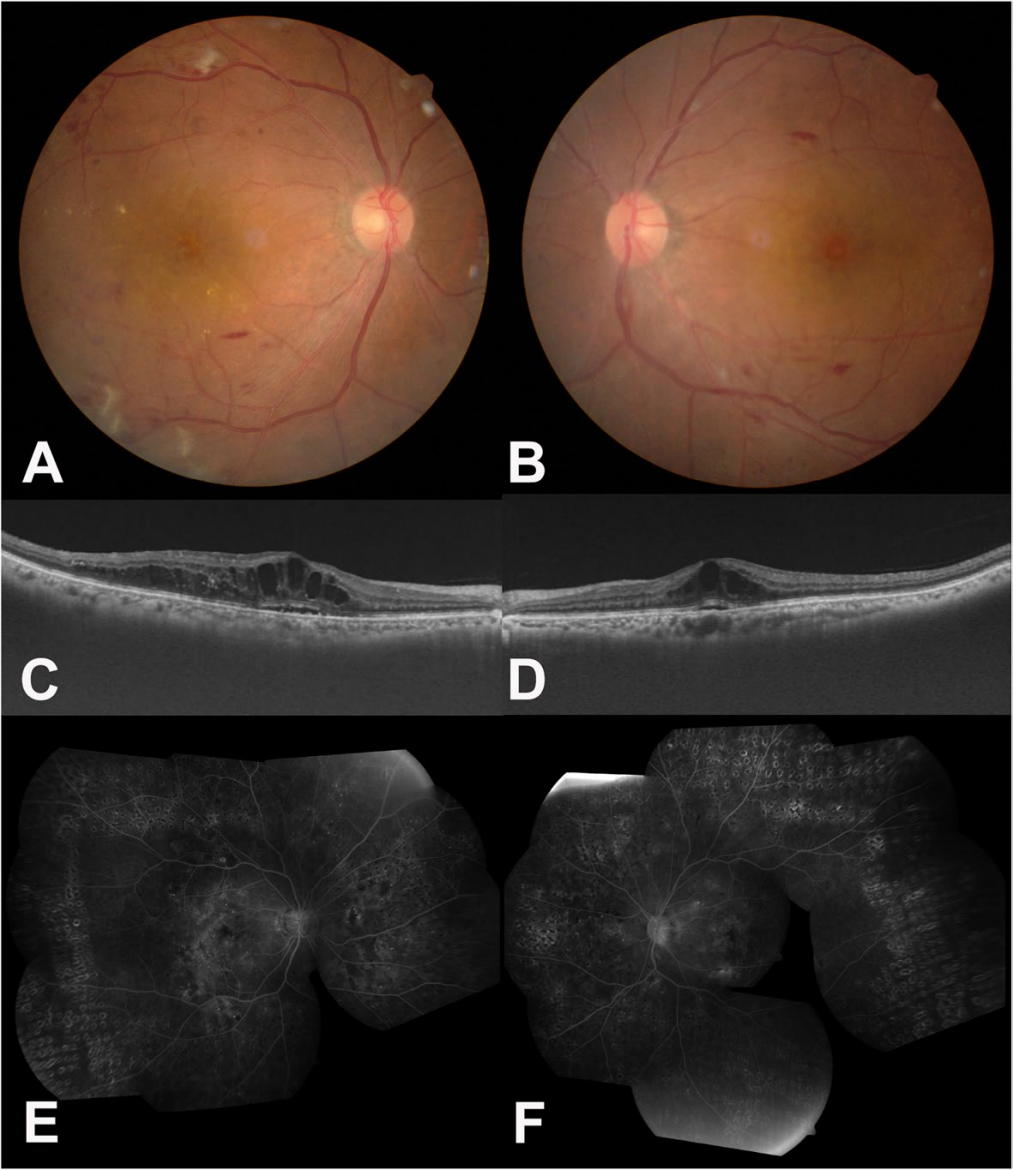
Ocular images at initial visit of patient in Case II. **A** and **B** Fundus photograph showed diabetic retinopathy with multiple dot hemorrhages and cotton wool spots in both eyes (**C** and **D**) Optical coherent tomography (OCT) showed severe macular edema in the right (**C**) and left (**D**) eyes. **E** and **F** Fluorescent angiography (FA) showed multiple laser scars at the peripheral retina and diffuse macular leakage

A 67-year-old woman was referred to our clinics for refractory diabetic macular edema (DME) in both eyes. Her visual acuity was 20/80 in the right eye and 20/40 in the left eye (OS). There were no cellular reactions in anterior chamber as well as vitreous in both eyes. Fundus photos showed retinal dot hemorrhages, cotton wool spots, and multiple laser scars in both eyes (Fig. 4A and B). OCTs showed macular edema in both eyes (Fig. 4C and D. FA demonstrated typical DME findings without definite signs of inflammation (Fig. 4E and F).

**Fig. 5:**
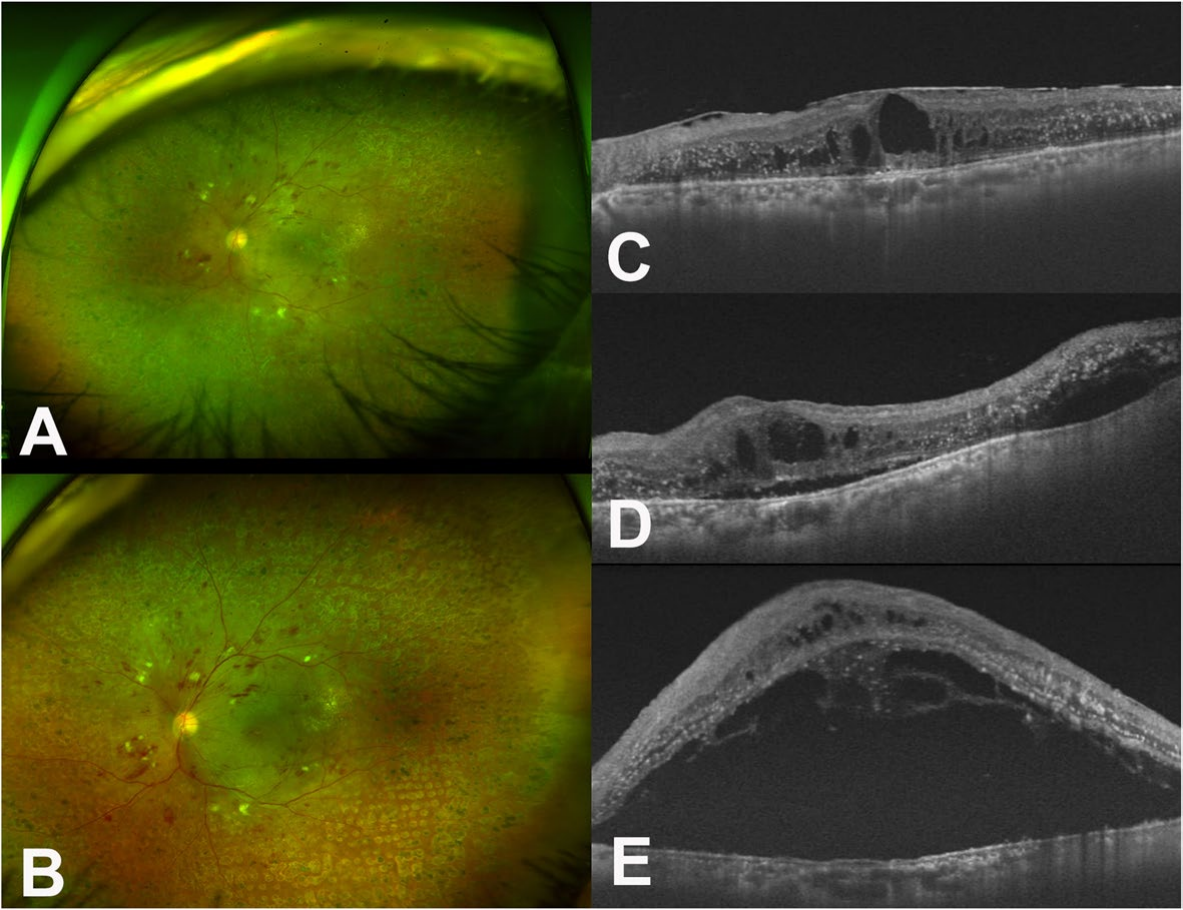
Series of ocular images of the left eye before penicillin therapy in Case II. **A** A fundus photograph on the day before vitrectomy showing multiple hemorrhages and cotton wool spots; **B** Fundus finding was not so much different from (**A**) other than edematous macula seven days after the vitrectomy (**C**) An optical coherent tomography (OCT) at the day before the vitrectomy showing epiretinal membrane (ERM) with intraretinal fluid (IRF). **D** There was nothing unique on OCT at post-operative Day 1 after the vitrectomy. **E** Massive subretinal fluid was noted seven days after the vitrectomy

The patient received four intravitreal bevacizumab injections, four intravitreal triamcinolone injections, and a dexamethasone implant in OS for eighteen months since the initial visit. Treatments with other anti-VEGF therapy was not possible due to insurance reasons. The DME had not been well controlled with bevacizumab, but while it had improved slightly following the triamcinolone injections and the dexamethasone implant, the DME did not completely resolve. Epiretinal membrane (ERM) thickened in OS (Fig. 5A and C), and responses to the therapies gradually weakened. Subsequently, vitrectomy was scheduled to remove the ERM.

Routine laboratory evaluations including a VDRL test had been done before the vitrectomy as part of pre-operative testing in our hospital. Positive VDRL was reported, and others were within normal limits. Patients denied any symptom related to syphilis and we did not find any ocular sign of syphilis on clinical ocular examination. FTA-ABS immunoglobulin G and M, and quantitative VDRL were tested to determine whether it was active syphilis infection. We explained those findings and our concern to the patient. Given that ocular findings were less likely to be ocular syphilis and the VDRL test tends to have a relatively high false positive rate, cataract surgery, vitrectomy, and internal limiting membrane (ILM) peeling were performed in OS on schedule before confirming the FTA-ABS test results.

At the end of surgery, triamcinolone 4.0 mg was injected into the posterior sub-tenon space. There were no abnormal findings the next day after the surgery (Fig. 5D). However, seven days after the surgical procedure, massive SRF was noted on fundus photo and OCT (Fig. 5B and E). The confirming test results were reported on the day the massive SRF was noted, showing positive FTA-ABS immunoglobulin G and high VDRL titer (1:16).

**Fig. 6:**
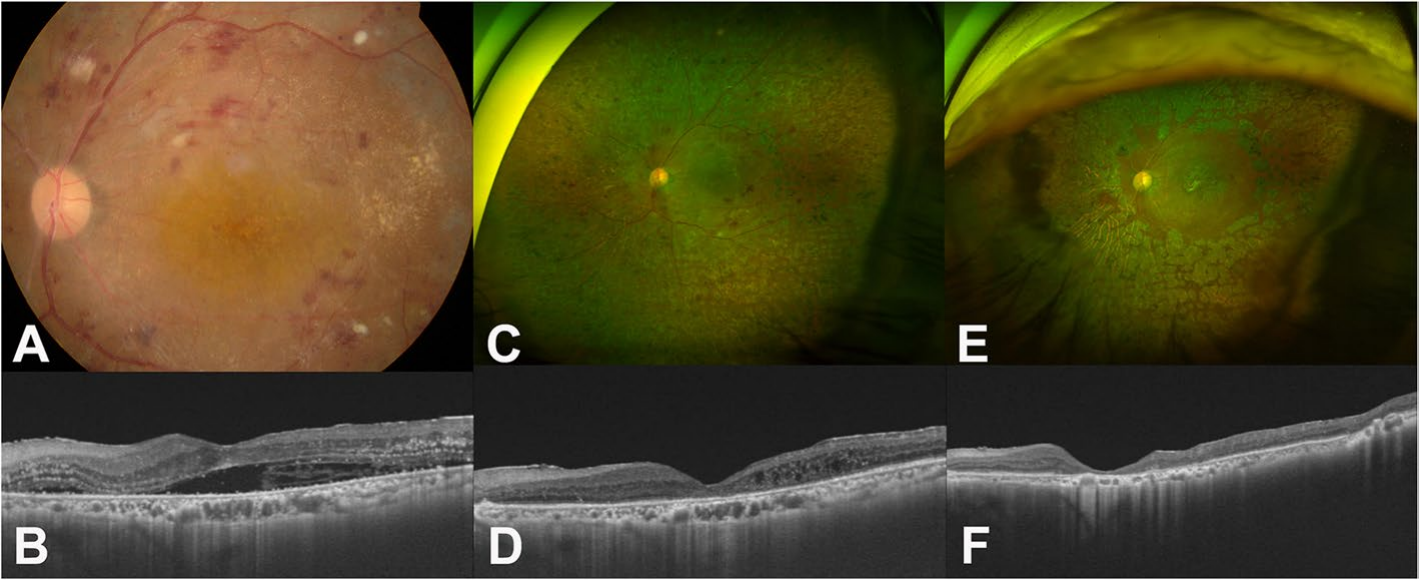
Series of ocular images of the left eye of the patient in Case II after penicillin therapy. **A** A fundus photograph was taken seven days after starting penicillin therapy (**B)** An optical coherent tomography (OCT) showed improvement of the submacular fluid seven days after starting penicillin therapy. **C** and **D** A wide-angle fundus photograph and OCT taken three months after the vitrectomy showed that the submacular fluid has resolved. **E** and **F** A wide-angle fundus photograph and OCT remained stable without any additional treatment for four years after the vitrectomy and intravenous penicillin therapy

The patient was referred to an infectious disease specialist and started on intravenous penicillin infusion were infused for two weeks. The SRF dramatically decreased one week after starting penicillin (Fig. 6A and B), and subsequently resolved (Fig. 6C and D) at three months after the surgery. The titer of VDRL decreased into 1:4 at three months after the treatment and then 1:2 at one year after the treatment. The macular edema has not recurred in OS without any further treatment for 48 months (Fig. 6E and F). In the right eye, the patient had received five anti-VEGF injections and four triamcinolone injections over the course of 18 months before starting the penicillin therapy. She received eight triamcinolone injections over 28 months afterwards. Responsiveness to the intravitreal injections has improved after the penicillin therapy in OD. The patient's best corrected visual acuity was 20/200 in the right eye and 20/250 in OS as of the most recent visit.

## Discussion

Syphilis infection has remained a medical challenge and one of the greatest medical masquerades, even though it can simply be confirmed by laboratory tests and effectively treated with penicillin [3]. Ocular infection and the corresponding inflammatory reaction are grouped under the term “ocular syphilis” [4–6]. Ocular syphilis presents in various forms, manifesting as keratitis [7], scleritis [8], chorioretinitis [4], acute syphilitic posterior placoid chorioretinitis (ASPPC) [9], or necrotizing retinitis [10]. The majority of uveitis specialists recommend syphilis testing for any patient who presents with uveitis, regardless of clinical presentation and risk factors [3].

The initial presentation of the two cases seems unlikely to be uveitis or ocular syphilis: they did not have any inflammatory signs on slit lamp examination, fundus examination, fundus photos, OCT, or FA, and they appeared to be more suggestive of neovascular AMD and DME, respectively.

There are few reported ocular syphilis cases having CNV [11–13] such as that in our first case. However, unlike the first case, the reported CNV was noted several years after the treatment of ocular syphilis [11–13]. On the other hand, we were not able to identify similar cases to our second patient in the literatures. A few reported cases showed that occult syphilis would become active following intravitreal steroid [14–16]. On the contrary, no inflammatory signs were noted even after the five intravitreal steroid injections in the second case.

The two cases were not suspected to be manifestations of ocular syphilis until the serologic results confirmed positivity for syphilis. The multiple hypo-fluorescent spots in late phase of ICGA led us to suspect uveitis in the first case based on the report of Mushtaq et al [17] showing multiple hypo-fluorescent spots in late phase ICGA were a common finding of ocular syphilis. We believe that ICGA might assist in detecting inflammatory signs even in cases where FA does not show typical findings of syphilitic uveitis.

As screening for syphilis is a routine laboratory evaluation before vitrectomy in our hospital, ocular syphilis was detected in the second case, even though the suspicion for ocular syphilis was low. Considering the fact that laboratory tests for syphilis are simple and relatively inexpensive, it may be worth expanding its indications beyond uveitis patients to include patients with refractory retinal diseases such as neovascular AMD and DME. We thought that it seemed false positive result given the ocular findings and the patient’s symptom. The second case gave a lesson that it would be dangerous if procedure performs in case with an undetermined VDRL result.

Given that improvements soon after starting intravenous penicillin therapy and VDRL titers decreased after the treatment, we diagnosed both cases as ocular syphilis with atypical presentations. On the other hand, it is possible that ocular syphilis might have co-existed with diabetic macular edema and neovascular AMD, respectively, making the retinal diseases refractory to anti-VEGF therapy.

Syphilitic infections, ocular or systemic, can be diagnosed with simple and inexpensive tests with high reliability and can be treated successfully with penicillin [5]. Laboratory tests for syphilis should remain to be for all patients diagnosed with uveitis [3]. The current two cases demonstrate that ocular syphilis can masquerade as refractory chronic retinal diseases. Therefore, laboratory evaluations for syphilis might be considered not only for uveitis but also for refractory retinal diseases. Indocyanine green angiography may also be beneficial in revealing occult ocular syphilis.

## Data Availability

All data generated or analyzed during this study are included in this published article.
